# 色谱技术在肝素结构分析中的研究进展

**DOI:** 10.3724/SP.J.1123.2022.07020

**Published:** 2023-02-08

**Authors:** Yilan OUYANG, Lin YI, Luyun QIU, Zhenqing ZHANG

**Affiliations:** 苏州大学医学院药学院，江苏 苏州 215127; College of Pharmaceutical Sciences，Suzhou Medical College of Soochow University，Suzhou 215127，China

**Keywords:** 色谱, 单糖组成分析, 二糖组成分析, 寡糖分析, 多糖分析, 肝素, 低分子量肝素, 综述, chromatography, monosaccharide composition analysis, disaccharide composition analysis, oligosaccharide analysis, polysaccharide analysis, heparin （Hp）, low molecular weight heparin （LMWHs）, review

## Abstract

肝素（heparin， Hp）是目前临床应用最为广泛的抗凝剂，是由重复二糖单元组成的多硫酸化酸性直链多糖。低分子量肝素（LMWHs）是以肝素为原料，经过化学或酶降解获得的相对分子质量相对较小的肝素衍生物，相对肝素，它们的出血副作用和免疫原性更小，皮下注射时生物利用度更高。肝素及低分子量肝素具有一系列结构特点，如相对分子质量偏大且有一定分布，多种糖残基同时存在，硫酸酯位置和数量呈现多样化，以及不同工艺产生的特殊残基的种类和含量不一等。该类药物结构的复杂性对分析方法提出了巨大的挑战，也限制了其质量控制提升、工艺优化、临床用药安全和新适应证拓展等。该文以色谱分析方法为中心，从结构分析的不同角度，包括单糖、二糖、寡糖、多糖的识别、组成分析和不同层次，系统地梳理和阐述近年来肝素类药物在色谱分析方法上的进展，并对这些方法的应用范畴、创新性、局限性等进行总结。该文将为肝素类药物的结构分析、质量控制提供较系统的方法学参考，为更多新方法开发提供思路，为更深入地研究肝素类药物结构、拓展其应用提供有力支撑。

## Abstract (en)

Heparin （Hp） is the most widely used anticoagulant drug in the clinics， with an annual global output of over 10 billion dollars. Hp， a member of the glycosaminoglycans （GAGs）， is prepared from porcine intestinal mucosa via extraction， separation， and purification. Hp is a linear polysaccharide with repeating disaccharide units. Low-molecular-weight heparins （LMWHs） are depolymerized from Hp via chemical or enzymatic degradation. Compared with Hp， LMWHs exhibit less bleeding side effect， milder immunogenicity， and higher bioavailability when injected subcutaneously. In general， Hps， including LMWHs， are high complex drugs with large molecular weights （MWs）， inhomogeneous MW distributions， and structural heterogeneity， including different degrees and locations of sulfonation， and unique residues generated from different production processes. Thus， developing efficient analytical methods to elucidate the structures of Hps and characterize or quantitate their properties is extremely challenging. Unfortunately， this problem limits their quality control， production optimization， clinical safety monitoring， and new applications. Research has constantly sought to elucidate the complicated structures of Hp drugs. Among the structural analysis and quality control methods of Hp currently available， chromatographic methods are the most widely studied and used. However， no literature thoroughly summarizes the specific applications of chromatographic methods in the structural analysis， manufacturing process， and quality control of Hp drugs. This paper systematically organizes and describes recent research progresses of the chromatographic methods used to analyze Hp drugs， including the identification and composition of monosaccharides， disaccharides， oligosaccharides， and polysaccharides. The applications， innovations， and limitations of these chromatographic methods are also summarized in this review. The insights obtained in this study will help production and quality control personnel， as well as drug researchers， obtain a deeper understanding of the complex structures of Hp drugs. This paper also provides a comprehensive reference for the structural analysis and quality control of Hps， proposes ideas for the development of new quality control methods， and lays a strong foundation for the in-depth structural elucidation of Hp drugs.

肝素（heparin， Hp）又称未分级肝素（UFH），作为抗凝剂使用已有近百年的历史，它是迄今为止最古老但依然在临床上广泛应用的抗凝药物^[1]^。肝素的主要临床适用症有动静脉血栓、肺栓塞、弥散性血管凝血等，可用于外科手术、血液透析等过程^[2]^。它作为销售额居全球首位的生化药物，2009年被列入世界卫生组织基本药物标准清单中，被认定为健康照护系统中最安全与最有效的药物之一^[3]^。可见，经过了近百年的发展历程，肝素在抗凝领域的地位依然举足轻重^[4]^。与此同时，肝素结构的异质性使一些肝素糖链可与血浆中多种蛋白分子结合。因此，肝素除了具有抗凝活性还展现了其他很多生物活性，如血脂调节、预防动脉粥样硬化、调节血管生长、抗肿瘤、抗炎、抗病毒等活性^[5⇓-7]^。特别是在治疗新冠肺炎（COVID-19）的初步结果中，肝素除了可以直接阻止病毒的侵袭外，还可以减轻病发过程中高凝状态级联反应激活的凝血障碍、血栓形成和器官损伤^[8⇓-10]^。

1916年约翰霍普金斯医学院的一名学生Jay McLean在促凝实验中意外从狗的肝脏中发现了具有抗凝作用的化合物，并认为是“肝磷脂”^[11]^。1918年，其导师Howell确证该化合物为碳水化合物而非磷脂类，随后改名为“肝素”^[12]^。从牛肺组织中提取的肝素在20世纪30年代进行了动物的体内抗凝血实验，瑞典Vitrum AB药厂在1936年首次完成了静脉注射肝素制剂的生产^[11]^， 1937年经人体试验确定为安全、有效且容易取得的抗凝血剂。1949年，Peter Moloney和Edith Taylor获得了低成本、高收率生产肝素的专利，使这种药物被广泛使用，第一代的未分级肝素产品就此诞生^[12]^。

肝素属于糖胺聚糖（GAGs）家族。它是由重复的氨基葡萄糖（GlcN）和葡萄糖醛酸（GlcA）或艾杜糖醛酸（IdoA）组成二糖单元通过1，4连接而成的直链多糖，其中GlcA与IdoA互为差向异构体；其重均分子质量约16000 Da，并有一定的相对分子质量分布；肝素糖链中硫酸酯基的位置和数量表现为多样性^[1]^，即肝素的结构呈现典型的微观不均一性，其基本结构示意图如图1所示^[13]^，其中含3位*O*-硫酸化GlcN的五糖序列是产生抗凝活性的关键结构，即与抗凝血酶Ⅲ（ATⅢ）的结合位点，该五糖序列为GlcNAc/NS6S （1→4） GlcA （1→4） GlcNS3S， 6S （1→4） IdoA2S （1→4） GlcNS6S（见图1）^[13⇓-15]^。

**图1:**
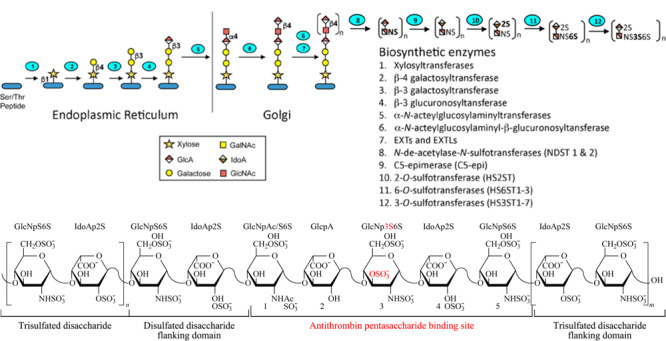
肝素生物合成与结构^[13]^

肝素结构的复杂性源于体内生物合成过程中参与的酶体系庞大、合成过程复杂。肝素的生物合成首先发生在肥大细胞的内质网中，如图1所示，在木糖基转移酶、*β*-3半乳糖基转移酶、*β*-4半乳糖基转移酶和*β*-3葡萄糖醛酸基转移酶先后作用下生成与核心蛋白端丝氨酸或苏氨酸连接的四糖连接域。随后以此四糖为起点在高尔基体中生成肝素，其中多种生物酶参与该过程，包括在*α*-*N*-乙酰氨基葡萄糖（GlcNAc）转移酶和*β*-葡萄糖醛酸转移酶作用下产生由重复的GlcA （1→4） GlcNAc二糖单元组成的糖链；在去*N*-乙酰基-*N*-磺基转移酶、糖醛酸C5异构酶（由GlcA转化成IdoA）、2位/6位/3位-*O*-磺基转移酶等作用下产生不同程度硫酸化修饰的肝素糖链^[16,17]^。由于部分酶异构体的存在及酶反应的不彻底性，生成的肝素具有以下几个结构特点：相对分子质量大且呈分散性分布、多种糖基组成以及硫酸酯基数量和位置多样^[17]^。

由此还可以看出，不同动物、相同动物的不同组织来源的肝素结构上可能存在差异，该推论近年来获得了大量研究结果的支持和验证^[18⇓⇓⇓-22]^。肝素最初被发现时的组织来源是狗的肝脏，但其并不适合大规模生产^[11]^。20世纪30~50年代，牛肺以原料充足、收益高的优点成为肝素生产的主要原料。之后肝素的提取源从牛肺转变为猪肠黏膜，原因有三：一是在处理、贮藏和提取大量牛肺组织的过程出现了肝素降解问题，产品质量受到影响^[23,24]^；二是随着香肠加工产业的兴起，猪肠黏膜作为香肠肠衣制作中的副产物，成为肝素制备的廉价原料来源^[4]^；三是20世纪80年代后期爆发的疯牛病，以牛肺为主的牛源肝素安全性受到质疑，美国市场上的牛源肝素均被生产商召回^[23,24]^。自此，全球主要药用肝素几乎全部以猪肠黏膜为原料。

在后续对肝素的抗凝机制研究中，研究者们发现了肝素发挥抗凝作用的关键：凝血蛋白酶FXa（Factor Xa，活化的X因子），它可以被肝素的核心五糖（GlcNAc/NS6S-GlcA-GlcNS6S3S-Ido2S-GlcNS6S）与ATⅢ形成的复合物拮抗^[25⇓⇓⇓-29]^。进一步的研究发现，18个糖以上的肝素糖链与ATⅢ形成复合物可以抑制凝血酶FⅡa（Factor Ⅱa，活化的Ⅱ因子），与出血副作用有关。于是研究者们试图在保留肝素核心五糖的基础上减小肝素糖链的长度，提高肝素的抗FXa/抗FⅡa活性比率^[30,31]^。由此，20世纪80年代，通过酶解或者化学降解等方法获得了不同的低分子量肝素（LMWHs）^[32]^。相比于未分级肝素，LMWHs具有更加优越的药代动力学特征，血浆半衰期更长，皮下注射后生物利用度更高，抗凝作用更为稳定^[33⇓-35]^。1996年，大型随机临床试验证实院外、不监测情况下，皮下注射LMWH与院内静脉注射普通肝素治疗血栓性疾病的疗效和安全性相当^[36]^。于是作为第二代的肝素产品，一系列LMWHs产品，包括依诺肝素钠（enoxaparin sodium）、那屈肝素钙（nadroparin calcium）、达肝素钠（dalteparin sodium）、亭扎肝素钠（tinzaparin sodium）等应运而生，正在逐步取代普通肝素用于血栓性疾病的预防和治疗^[32,37]^。

这些LMWHs在保留了肝素原有结构特点的基础上，由于制备方法不同，出现了一系列相应的特征结构。如苄基化反应后，在碱性条件下降解制备的依诺肝素钠部分糖链还原端产生1，6脱水葡萄糖胺或甘露糖胺（1，6-an. A/M），部分非还原端产生4，5脱水糖醛酸（4，5-unsaturated uronic acid， ΔUA）^[31]^；通过亚硝酸降解制备的那屈肝素钙或达肝素钠在还原端产生了2，5脱水甘露醇结构（2，5-anhydromannitol， 2，5-AM. ol）^[38]^。这些特征残基的产生，无疑进一步增大了LMWHs糖链结构的复杂性。

以肝素核心五糖为模板全合成的磺达肝癸钠是肝素类药物第三代产品的代表，也是第一个单一结构的肝素产品^[39]^。相较于第一代的未分级肝素和第二代的低分子量肝素，磺达肝癸钠能通过皮下注射吸收，具有更高的生物利用度，出血副作用更低。然而，磺达肝癸钠也有着不可忽视的缺陷，首先是在应用上磺达肝癸钠无法被鱼精蛋白拮抗，即缺少解毒剂，且其肾毒性限制了肾功能障碍人群的使用^[40]^；其次，磺达肝癸钠作为化学合成的五糖，合成路径很长，产率仅为0.1%，生产成本极高。这些问题导致该产品临床使用率远不及LMWHs。

百年肝素经历了几次里程碑式的发展，奠定了今天全球以LMWHs为主，未分级肝素和合成肝素占一定份额的市场格局。受饮食习惯和宗教因素的影响，中国成为肝素原料药最大的出口国，全球超过50%的猪肠黏膜肝素原料药来自中国^[4]^。近年来，中国肝素科研投入巨大，多家企业已经成为LMWHs原料药和制剂的全球供应商。然而，肝素类产品的发展并不是一帆风顺的。2008年肝素污染事件中，被肝素类似物过硫酸软骨素（OSCS）污染的肝素产品，超出了当时的质控检测范畴，最终流入市场，导致超过200名使用相关产品的患者死亡^[41⇓-43]^。该事件就像一记警钟，提醒着我们肝素类产品结构的复杂性、相关分析方法的局限性、质量控制深入研究的紧迫性，只有不断深入发展和完善相关研究，才能确保肝素药物临床应用的安全性以及相关新产品开发的可靠性^[44]^。

2008年肝素污染事件发生以后，巨大人力物力投入到肝素类药物的结构、分析方法、质量控制等研究中，大量的相关分析方法，特别是色谱分析方法应运而生。它们大多围绕着肝素类药物的糖基组成、二糖组成、重要结构域组成、寡糖组成、多糖组成等来建立，其中离子色谱检定肝素中半乳糖胺限量、强阴离子色谱（SAX）测定依诺肝素钠脱水还原端含量等方法已经被收入药典中；用于低分子量肝素寡糖谱分析的分子排阻色谱结合高分辨质谱联用技术（SEC-MS）、用于那屈肝素钙和达肝素钠中脱水还原端含量分析的离子色谱-质谱联用技术（IC-MS），以及多中心切割二维液相色谱-质谱联用技术（MHC 2D-LC-MS）已成功应用到LMWHs药物的一致性研究中；SAX进行聚糖分析检测肝素中OSCS、硫酸皮肤素（DS）等不纯物含量的方法在产品工艺和质量提升过程中得以应用^[45⇓-47]^。

本文将从单糖、二糖、寡糖和多糖等多个维度，对当前肝素结构分析中应用的色谱分离方法的优势和不足进行梳理和对比，针对色谱技术在肝素类药物质量控制中的应用做系统的总结，为肝素类药物的质量控制方法提供参考，并为肝素类药物精细结构的阐释提供线索，推动肝素类药物安全有效可控地应用。

## 1 单糖组成分析

肝素是由GlcN和IdoA或GlcA形成的重复二糖组成。肝素中常见的天然杂质DS和硫酸软骨素（CS）与肝素同属GAGs，它们是由氨基半乳糖（GalN）和IdoA或GlcA形成的重复二糖组成的硫酸化直链多糖^[1]^。2008年肝素污染事件中的OSCS，是人工合成的CS的过硫酸化衍生物，单糖组成同样为GalN和GlcA。据此，单糖组成分析可以有效鉴别和定量检查肝素以及可能存在的DS、CS或OSCS。

进行多糖的单糖组成分析时，大多需要将单糖释放，酸水解是其中主要的手段。理想的水解条件可以在完全释放糖基的同时，尽量减少单糖结构的破坏。多项针对肝素酸水解条件优化的研究包括：2~4 mol/L三氟乙酸（TFA）在100~110 ℃下降解8~24 h^[48⇓-50]^， 3~5 mol/L盐酸在100 ℃下降解6 h^[51]^，微波100 W 10 min^[52]^以及采用聚合酶链式反应（PCR）设备在30 min内对多达96个样品进行酸解处理^[53]^。其中盐酸水解是被《美国药典》（USP）肝素钠半乳糖胺限量检查分析采纳的方法^[54]^，该方法稳定有效可控。

自然界中单糖的种类有数十种，它们均为多羟基醛或酮或醛酸，亲水性强，无特征紫外吸收，大多存在差向异构体。这为综合性单糖分析方法提出了不小的挑战。最早出现的单糖分析方法为纸色谱法^[55]^，以及薄层色谱法^[56]^，但它们的分辨率和灵敏度均较低，已无法满足现代化综合分析的需求。本文重点梳理了肝素类药物单糖现代化色谱分析方法以及潜在可能用于肝素类药物单糖分析的方法。

### 1.1 离子色谱结合脉冲安培检测法（IC-PAD）

利用IC-PAD的单糖分析过程中，碱性流动相使单糖的羟基电离，并与阴离子色谱柱有不同程度的结合和保留，在流动相洗脱下达到高效分离的目的，同时，PAD检测器利用单糖半缩醛的氧化还原性成功实现了高灵敏度和特异性检测。2012年，Zhang等^[45]^建立了一种IC-PAD方法对16种单糖一次性系统分析，包含多种中性糖、氨基糖和酸性糖等（见图2）。研究者利用该方法有效对比了肝素、CS、DS等GAGs单糖组成的差异，同时，确定了较低的定量限（12.5×10^-3^ nmol， ~2.5 ng）和较宽的线性范围。USP收录了离子色谱结合脉冲安培检测方法用于肝素的GalN限量检查（含量不超过所有氨基糖总量的1%），确保肝素产品中只含有极低的GalN相关的CS、DS，也包括OSCS。在此基础上，Yi等^[57]^应用该方法成功实现了那屈肝素钙和达肝素钠糖链末端结构2，5-AM. ol的定性定量分析，成为该方法在肝素类药物分析中又一个有意义的应用。

**图2:**
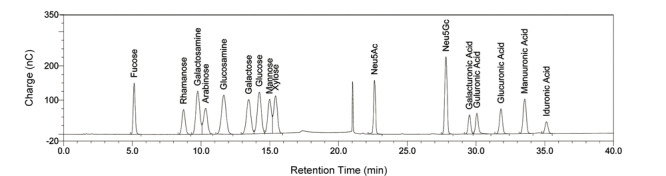
IC-PAD分析16种单糖的色谱图^[45]^

### 1.2 衍生化法

柱前衍生可以有效改变单糖的色谱行为，同时衍生化后可以进行相应的紫外或荧光检测。常用的单糖衍生化试剂有1-苯基-3-甲基-5-吡唑啉酮（PMP）、2-氨基苯甲酰胺（2-AB）、2-氨基吡嗪（2-AP）、4-氨基苯甲酸乙酯（ABEE）等^[51⇓-53]^。2021年，Wang等^[58]^通过合成新的衍生物*O*-（4-甲氧基-d3苄基）-羟胺盐酸盐（4-MOBHA·HCl），使12种单糖（包含肝素类药物含有的GlcN、GlcA，以及相关GalN）在C18色谱柱上基线分离，该衍生物还大大提高了检测灵敏度，定量限低至0.25~3.00 fmol/L，可以应用于各种生物样本的分析。这些单糖经过衍生化后的色谱分析方法，因仪器适配性和色谱稳定性高，有着广泛的应用以及潜在的肝素类药物单糖分析能力。当然，衍生化过程相对复杂、反应效率不同等问题可能会导致结构信息丢失或不准确，一定程度上限制了其应用。

表1汇总了用于或者可能用于肝素类药物单糖分析的方法，包含样品前处理、色谱方法等。

**表1:** 单糖组成分析方法

No.	Derivatization reagent	Method	Parameters	Analytes	Ref.	Published year
1	PMP	RPLC-UV	LOD：0.04-1.6 μmol/L；LOQ：0.15-1.6 μmol/L；mass recovery：92%-100%；RSD：0.3%	10 sugars	[51]	2016
2	PMP	RPLC-MS（MRM）	LOD：0.056-5.6 fmol/L；LOQ：0.5-10 ng/mL；RSD：6.0%	16 sugars	[53]	2017
3	2-AB and 2-AP	RPLC-UV	LOD：1.2-11 nmol/L；LOQ：4-36 nmol/L；repeatability：2%-9%；inter-day repeatability：3%-9%	10 sugars	[52]	2021
4	d3-4-MOBHA·HCl	RPLC-MS（MRM）	LOD：1.2-11 nmol/L；LOQ：0.25-3 fmol/L；mass recovery： 85%-110%	12 sugars	[58]	2021
5	no	IC	LOD：1.0 ng；LOQ：2.5 ng	16 sugars	[45]	2012

PMP：1-phenyl-3-methyl-5-pyrazolone；2-AB：2-amino benzamide；2-AP：2-amino pyrazine；d3-4-MOBHA·HCl：*O*-（4-（methoxy-d3）benzyl）hydroxylamine hydrochloride.

## 2 二糖分析

重复二糖是GAGs的特征性结构。肝素类药物重复二糖单元中存在不同硫酸酯基位置和数量差异，这些有着不同硫酸酯基位置和数量的二糖比例可能随着动物来源、制备工艺、厂家甚至批次的不同而不同^[59]^。于是，彻底酶解后的二糖分析是GAGs，特别是肝素类药物结构解析过程中不可或缺的内容之一。肝素糖链通过肝素裂解酶Ⅰ、Ⅱ和Ⅲ彻底酶解可以产生常见二糖、糖链末端四糖连接域、肝素酶不识别特殊结构等几十种产物^[60]^。裂解酶降解释放的肝素类药物二糖或者寡糖的糖链非还原端均带有4，5位双键，与糖醛酸的羧基形成共轭，在约232 nm处呈现特征吸收，结合不同分离机制和适宜的检测手段完成它们的定性定量分析可以实现肝素类药物基本单元组成的重要表征。

### 2.1 强阴离子交换色谱-紫外法

SAX是一种以高浓度非挥发性盐为流动相，根据肝素二糖或寡糖的离子强度进行分离的色谱方法。结合UV检测器，SAX体现出了分辨率高、稳定性好、定量准确等特点。USP^[61]^和《欧洲药典》（EP）^[62]^在利用二糖分析检测依诺肝素钠糖链末端1，6-an. A/M含量过程中均采用了SAX-UV方法。在该方法中，依诺肝素钠经过混合肝素裂解酶彻底酶解产生十几种酶解产物，这些产物主要为酶解释放出来的二糖结构，同时也包括依诺肝素钠糖链含有1，6-an. A/M的末端结构。酶解释放出来的二糖含有完整还原端，被硼氢化钠还原后色谱保留时间系统性前移，而含有1，6-an. A/M末端结构的酶解产物无法被还原，色谱保留时间不变，通过这种差异所有含有1，6-an. A/M末端结构的酶解产物均被识别并进行了归一化法定量测定。该方法得益于SAX-UV较高分辨率和较好稳定性。

2015年Mourier等^[63]^利用SAX方法对不同来源和批次的肝素彻底酶解产物进行了定量的指纹图谱研究，其中包含常规二糖、3-*O*硫酸化的二糖和四糖、糖链末端丝氨酸残基等二十几种酶解产物（见图3a）。在分析过程中，由于实际样品的复杂性以及含量较小组分标准品的缺失，有些归属出现了不明确和不准确的情况。

**图3:**
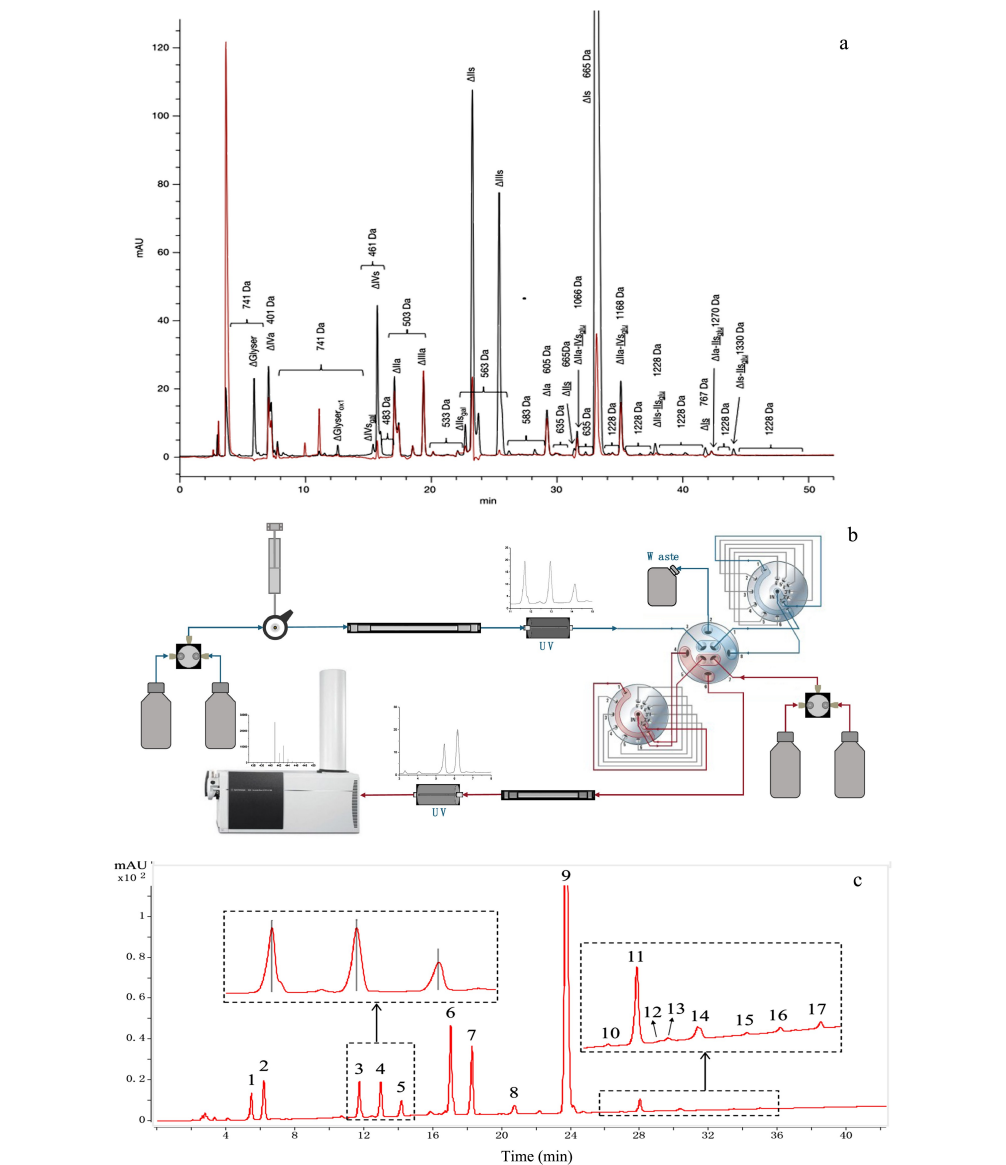
强阴离子交换色谱法定量分析肝素彻底酶解产物^[63,65]^

在线质谱是解析未知峰的有力手段，但SAX以非挥发性盐为流动相，无法实现与质谱的在线联用。为了突破SAX与质谱联用的技术壁垒，2016年Miller等^[64]^开发了以挥发性盐（碳酸氢铵，VS）为流动相，在十六烷基三甲基铵（CTA）键合的C18色谱柱上分离肝素二糖和寡糖的方法。VS-CTA-SAX方法实现了SAX与质谱兼容并初步完成了肝素二糖及寡糖的分离，但整体分辨率有所下降，同时碳酸氢铵的可分解性对该方法的稳定性提出了挑战。2021~2022年Chen等^[65,66]^开发了多中心切割（multiple heart cut， MHC）二维液相色谱与质谱联用（2D-LC-MS）的方法，SAX-UV作为第一维色谱对肝素类药物彻底酶解产物进行有效分离，之后指定色谱峰自动进入第二维分子排阻色谱（size-exclusion chromatography， SEC）在线脱盐后进入MS进行定性分析（见图3b和3c）。利用该方法研究人员对17种肝素彻底酶解产物、15种依诺肝素钠彻底酶解产物、20种那屈肝素钙彻底酶解产物进行了定性定量分析。该方法继承了传统SAX的稳定性、准确度和分辨率，同时实现了在线质谱定性分析，为肝素类产品的质量控制和构效关系研究提供了有力的分析工具。

### 2.2 反相离子对色谱-质谱法（IPRP-MS）

肝素类药物的二糖及寡糖等由于具有极强的亲水性，在常规的反相色谱柱中较难保留。离子对试剂的加入使这些被分析物在流动相中与正电性的有机离子对试剂生成一定亲脂性的中性分子，从而增强肝素二糖在色谱中的保留，改善分离效果^[67]^。离子对试剂的空间体积和疏水性在肝素二糖或寡糖的解析中发挥了复杂的分析物竞争作用，因此对于离子对试剂的选择和浓度的确定需要优化^[68]^。在肝素二糖或寡糖分析时，常用的离子对试剂有三丁胺（TBA）和乙酸铵、丁胺（BTA）和乙酸铵、戊胺（PTA）和六氟异丙醇（HFIP）等^[67⇓⇓-70]^。虽然离子对试剂或者有机相本身的紫外吸收影响了酶解糖链紫外检测，但挥发性流动相与MS有较好的兼容性，质谱的检测既有较高灵敏度又可以实现定性分析的目的。2008年Zhang等^[67]^使用微流液相以TBA和乙酸铵为离子对试剂，离子阱MS为检测器，并利用^13^C、^15^N标记后的二糖作为内标校正MS离子化效率带来的差异，实现准确定量分析，定量限低至2 ng（见图4）。

**图4:**
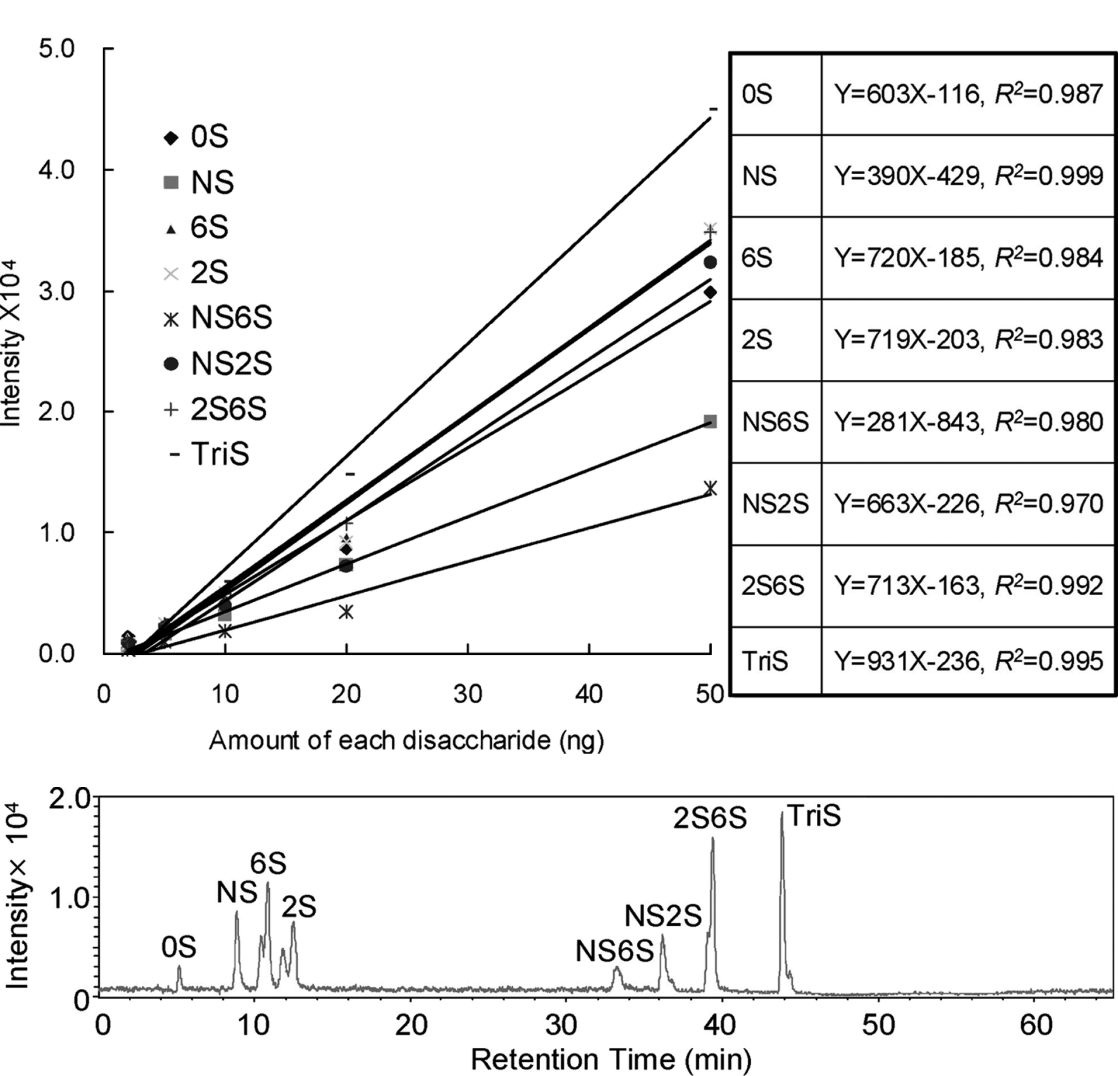
微流反相离子对色谱-质谱法（Mf-IPRP-MS）对8个肝素二糖的定量分析结果^[67]^

2015年Xu等^[71]^利用IPRP-MS法对依诺肝素钠和那屈肝素钙的部分酶解产物从二糖（dp2）至十糖（dp10）约两百多个寡糖组分进行定性定量分析，其中离子对试剂为PTA和HFIP。IPRP对肝素类药物二糖和寡糖均有较好的分离效果，色谱分辨率较高，同时较好的MS兼容性使该方法能对未知或不常见的组分定性分析，是一种高效、便捷的肝素类药物结构分析方法。然而，离子对试剂的加入也产生了一些弊端，如离子对试剂与被测化合物发生不同程度的加合，产生多种加合产物，一定程度上增加了质谱解析的难度^[68]^；离子对试剂容易与色谱柱填料发生不可逆结合，导致色谱柱效率降低，色谱柱使用寿命缩短；离子对试剂还在MS中存在严重的残留现象，随着使用次数的增加离子对试剂在MS中的响应明显增加，对质谱的数据采集产生影响。这些弊端限制了该方法更为广泛的使用。

### 2.3 亲水相互作用色谱-质谱法（HILIC-MS）

HILIC也可称为“含水正相色谱”，是一种集正相、离子交换等多种分离机制于一体的混合色谱模式，适用于强极性化合物的分离。该法通常以含水流动相作为洗脱剂，被测物的亲水性越强，在色谱上的保留越强，非常适合亲水性强的肝素类药物二糖分析^[72]^。HILIC的流动相是水相缓冲盐与有机相混合，一般选用挥发性盐如甲酸铵、乙酸铵等，水相的比例约3%~40%，因此与MS具有较好的兼容性。2016~2017年Sun等^[60,73]^利用HILIC分离并采用多反应监测（MRM）模式定性定量分析依诺肝素钠和那屈肝素钙彻底酶解产物。该方法的分离度有限，但借助被测物在MS中质荷比（*m/z*）以及碎片离子的不同，实现了30多种糖结构的分析（见表2）。2012年Gill等^[74]^利用HILIC-MS对GAG酶解产生的具有Δ4，5不饱和糖醛酸的二糖和亚硝酸降解产生的饱和二糖（含2，5-AM. ol）进行定量分析，该方法实现了简单的LC-MS平台确定GAG中的二糖谱。HILIC的分辨率较差，UV背景高且不稳定，但与MS兼容性好，其与MS联用极大提高了该方法的适用性，在肝素类药物结构解析过程中有良好的应用。

**表2:** 不同分析方法获得的肝素或低分子量肝素的二糖组成

No.	Composition	SAX- UV^Hep[63]^	SAX- SEC^Hep[65]^	SAX- SEC^Eno[66]^	SAX- SEC^Nadro[66]^	IPRP- MS^Eno[70]^		HILIC- MS^Nadro[72]^		HILIC- MS^Eno[60]^
1	ΔU-GlcNAc	1	1	1	1	1		1		1
2	ΔU-GlcNS	1	1	1	1	1		1		1
3	ΔU-GlcNAc，6S	1	1	1	1	1		1		1
4	ΔU2S-GlcNAc	1	1	1	1	1		1		1
5	ΔU-GlcNS，6S	1	1	1	1	1		1		1
6	ΔU2S-GlcNS	1	1	1	1	1		1		1
7	ΔU2S-GlcNAc，6S	1	1	1	1	1		1		1
8	ΔU2S-GlcNS，6S	1	1	1	1	1		1		1
9	ΔGalA-GlcNS	1	0	0	0	0		1		1
10	ΔGalA-GlcNS，6S	1	0	0	0	0		1		1
12	IdoA2S-GlcNS	1	0	0	0	0		1		1
13	U2S-GlcNS or U-GlcNS，6S	0	0	1	0	0		0		0
14	U2S-GlcNAc or U-GlcNAc，6S	0	0	0	1	0		0		0
15	IdoA2S-GlcNS，6S	1	0	0	0	0		1		1
16	ΔU2S-GlcN，6S	0	0	0	0	0		1		1
17	ΔU2S-GlcN	0	0	0	0	0		1		1
18	ΔU-GlcN，6S	0	0	0	0	0		1		1
19	ΔU-GlcN	0	0	0	0	0		0		1
20	ΔGlyser	1	1	1	1	0		1		1
No.	Composition	SAX- UV^Hep[63]^	SAX- SEC^Hep[65]^	SAX- SEC^Eno[66]^	SAX- SEC^Nadro[66]^		IPRP- MS^Eno[70]^		HILIC- MS^Nadro[72]^	HILIC- MS^Eno[60]^
21	ΔGlyser_ox1_	1	0	0	0		0		1	1
22	ΔGlyser_ox2_	1	0	0	0		0		0	0
23	Other non-endogenous derivatives ΔI_SO3_	1	0	0	0		0		0	0
24	Tetrasaccharide A ΔI_S_-I_2SO3_	1	0	0	0		0		0	0
25	Tetrasaccharide B ΔI_2SO3_-I_sid_	1	0	0	0		0		0	0
26	ΔU-GlcNAc，6S-GlcA-GlcNS，3S	1	0	0	0		0		0	0
27	ΔU-GlcNAc，6S-GlcA-GlcNS，3S，6S	1	1	1	1		1		1	1
28	ΔU-GlcNS，6S-GlcA-GlcNS，3S，6S	1	1	1	0		0		1	1
29	ΔU2S-GlcNAc，6S-GlcA-GlcNS，3S，6S	1	0	0	0		0		1	0
30	ΔU2S-GlcNS，6S-GlcA-GlcNS，3S，6S	1	0	0	0		0		1	0
31	GlcNS-IdoA2S-GlcNS，6S or GlcNS，	0	0	1	0		0		0	0
	6S-IdoA2S-GlcNS									
32	GlcNS，6S-IdoA2S-GlcNS，6S	1	0	0	0		0		1	1
33	ΔU-GlcNS，6S-HexA	0	0	0	0		0		1	1
34	ΔU2S-GlcNAc，6S-HexA	0	0	0	0		0		0	1
35	ΔU2S-GlcNS，6S-HexA	0	0	0	0		0		1	1
36	ΔU2S-GlcNS，6S-HexA2S	0	0	1	0		0		1	1
37	ΔU2CS-GlcNS，6S	0	0	0	0		0		1	1
38	GlcNS，6S-U2CS-GlcNS or GlcNS-U2CS-GlcNS，6S	0	0	0	1		0		0	0
39	ΔUA2S-GlcNS，6S-GlcA-2，3-anhydro-GlcNS	0	0	0	0		0		1	1
40	ΔUA-GlcNS-HexA2S，3S-GlcNS	0	0	0	0		0		1	1
41	3-*O*-S Δdp2	0	0	0	0		0		1	0
42	3-*O*-S Δdp4（3S，1Ac）	0	0	0	0		0		1	0
43	3-*O*-S Δdp4（4S，0Ac）	0	1	1	0		0		1	0
44	3-*O*-S Δdp4（4S，1Ac）	0	2	0	0		0		0	0
45	3-*O*-S Δdp4（5S，0Ac）	0	3	0	0		0		0	0
46	ΔU- Mnt6S	0	0	0	1		0		1	0
47	ΔU2S- Mnt6S	0	0	0	1		0		1	0
48	GlcNS，6S-U2S-Mnt6S	0	0	0	1		0		0	0
49	ΔU2S-GlcNS-U-Mnt6S	0	0	0	0		0		1	0
50	ΔU2S-GlcNAc-U2S-Mnt6S	0	0	0	1		0		0	0
51	ΔU2S-GlcNS-U2S-Mnt6S	0	0	0	1		0		1	0
52	ΔU2S-GlcNS，6S-U2S-Mnt6S	0	0	0	1		0		1	0
53	MntΔdp5（5S，1Ac）	0	0	0	1		0		0	0
54	MntΔdp6（6S，1Ac）	0	0	0	1		0		0	0
55	ΔU-1，6-anhydroGlcNS	0	0	0	0		0		0	1
56	ΔU-1，6-anhydroManNS	0	0	0	0		0		0	1
57	ΔU2S-1，6-anhydroHexNS	0	0	0	0		2		0	1
58	GlcNS，6S-U2S-1，6-anhydroHexNS	0	0	1	0		0		0	0
59	ΔU2S-GlcNS，6S-U2S-1，6-anhydroManNS	0	0	0	0		1		0	1

Hep：heparin sodium；Eno：enoxaparin sodium；Nadro：nadroparin calcium；Mnt：2，5-AM. ol；SEC：size-exclusion chromatography；HILIC：hydrophilic interaction chromatography；ΔU2S-GlcNAc，6S：unsaturated disaccharide with 2-*O*-sulfated uronic acid and *N*-acetylated，6-*O*-sulfated glucosamine；3-*O*-S Δdp4（3S，1Ac）：unsaturated tetrasaccharide with 3-*O*-sulfated glucosamine（total three sulfo groups，one acetyl group）；1：detected；0：no detected.

### 2.4 毛细管电泳法（CE）

面对肝素类药物二糖这种高电荷物质的分析，CE有着高分辨和高灵敏度的独特优势。传统CE因电解质缓冲液多为磷酸缓冲盐等不挥发性盐，无法直接和MS串联，因此传统CE常与紫外或荧光检测器串联使用。2017年Zhang等^[75]^采用CE-UV法对LMWHs中具有不同ATⅢ结合活性组分进行二糖分析（见图5a）。该研究中，在峰归属的过程中利用电泳迁移率和荷质比呈正比的原理对含3-*O*硫酸化的3个四糖进行归属，完成了高分辨率的定性定量分析。近些年随着CE与MS联用技术的发展，CE分离过程中的未知峰实现了在线结构解析。2016年Sun等^[76]^利用CE-MS联用技术对不同硫酸化程度的肝素二糖进行定性定量分析，该方法线性良好，定量限低至6 ng/mL。方法中CE可以与MS串联是源于可挥发性背景电解质（NH_4_HCO_3_）和一款新颖的电动泵驱动的CE-MS离子源（CMP EMASS-II CE-MS），该离子源提供低流速鞘流液（50%~70%甲醇水溶液含0.1%~1%甲酸）和雾化电压，但可挥发性的电解质降低了CE对肝素二糖的分辨率，导致该方法仅能对含有不同硫酸基团的肝素二糖进行分离，一定程度上限制了该方法的应用。2019年Ouyang等^[77]^利用等电点聚焦电泳（cIEF）与MS联用对肝素二糖进行分离，这是首次以等电点为分离依据分离酸性二糖，该研究中同样使用了外加离子源（见图5b）。该方法对肝素二糖分离的分辨率有所提高，但是目前还未能实现全部二糖的基线分离，还有待新型填充毛细管的开发。总之CE的超低流速、高灵敏度是该方法的优势，相信随着毛细管技术的发展，更多应用将随之产生。

**图5:**
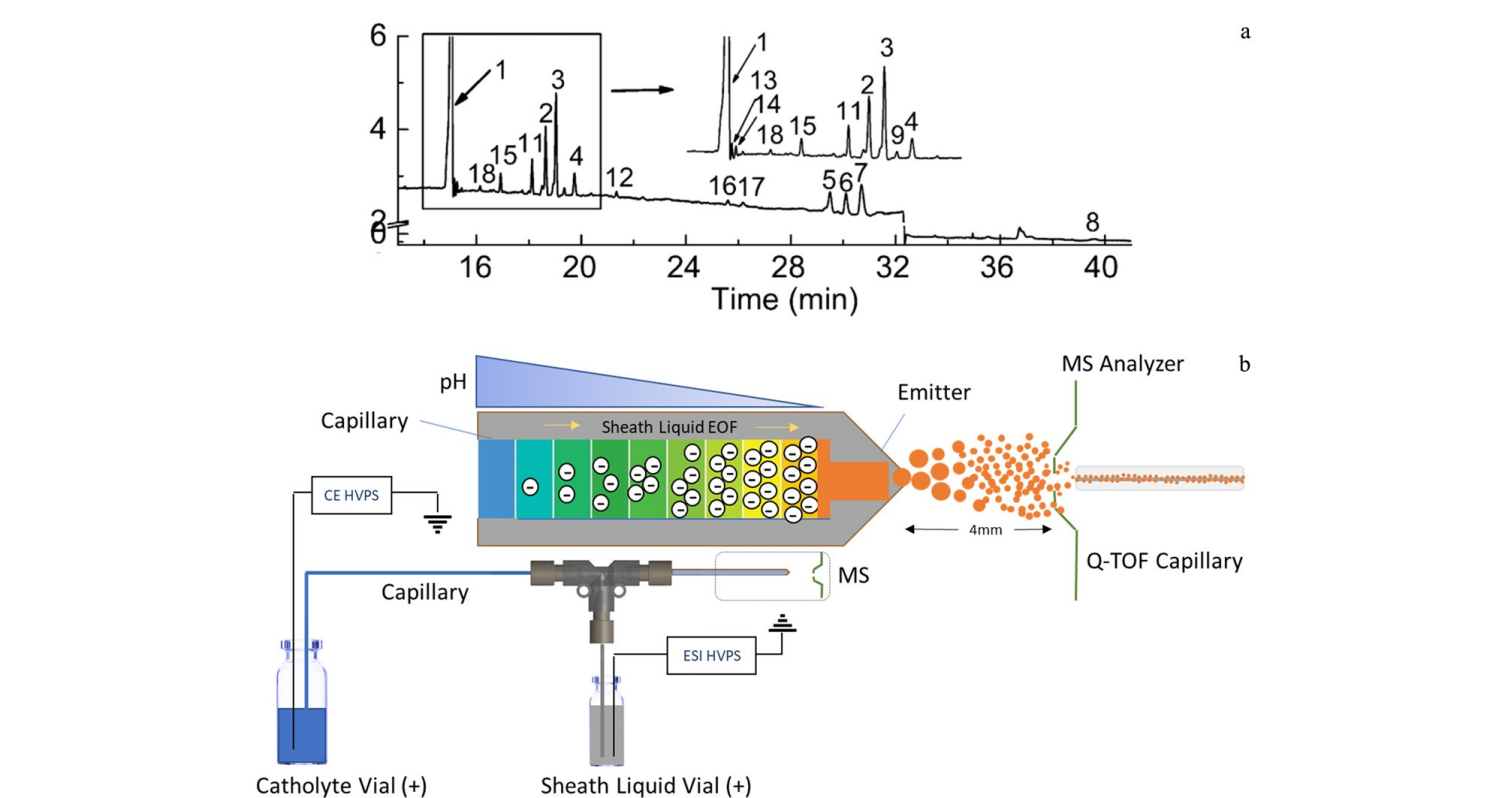
CE-UV法定量分析依诺肝素酶解产物^[75,77]^

### 2.5 衍生化方法

与单糖分析类似，通过柱前衍生可以改变肝素类药物二糖的色谱行为，同时引入的发光基团使检测手段多样化，检测灵敏度进一步提高^[69,78⇓-80]^。肝素、CS和透明质酸（hyaluronic acid， HA）的17种二糖经过2-氨基吖啶酮（AMAC）衍生化后，可在RP^[79]^、HILIC^[80]^色谱柱上以及CE^[81,82]^上实现完全分离，借助FID或MS实现准确、快速定性定量分析。2014年Ucakturk等^[83]^采用CE-FID实现了2-氨基吖啶酮衍生化的硫酸乙酰肝素（HS）、CS、硫酸角质素（KS）以及HA等19种GAG二糖的基线分离，在一次实验过程中对所有GAG相关二糖进行快速、准确、超高灵敏的定性定量分析。2021年Wang等^[84]^利用6-氨基-*N*-（2-二乙氨基）乙基喹啉-2-甲酰胺（AMQC）衍生，在MRM质谱模式下定量分析了肝素二糖，相较于AMAC衍生灵敏度增大了20~320倍。这些方法的灵敏度高，特异性强，是生物样本如细胞、组织、体液中GAG二糖定性定量分析的常用方法。表2系统地总结了不同二糖分析方法下可以检测到的肝素类药物中的结构片段，一定程度上体现了这些方法的灵敏度和分辨率。

## 3 寡糖分析

肝素类物质是一系列线性硫酸多糖和寡糖的组合，它们的相对分子质量，即糖链聚合度，在一定范围内分布，同时每一个聚合度下又存在糖基组成、硫酸酯基数量或位置不同的糖链序列分布。对未分级肝素部分酶解所得寡糖以及对LMWHs寡糖进行系统分析是肝素类药物结构确认、构效关系研究以及结构改造过程中重要的支撑性工作。然而，鉴于上述提到的糖类物质结构特点以及肝素类药物寡糖相对分子质量分布和结构的微不均一性特点，针对这类物质的分析难度要远大于单糖和二糖分析。单独的色谱分析往往无法满足肝素类寡糖分析的要求^[85]^，色谱-质谱联用技术成为肝素类寡糖分析的主要手段，其中常用的色谱方法有IPRP、HILIC、SEC、CE等^[85⇓⇓-88]^。近些年随着多维色谱技术的不断发展，研究者已经开始尝试将多维色谱技术应用于肝素类寡糖的分析当中，呈现出了较好的效果。同时针对肝素类寡糖的质谱数据库以及自动化质谱归属软件也在不断发展，一定程度上解决了该类物质色谱-质谱联用数据复杂、人工解析繁复的问题。

### 3.1 IPRP-MS

如二糖分析中所述，IPRP对肝素类二糖有良好分离效果，这些方法可以拓展到肝素类寡糖分析，但分辨率相对降低。2009年Catalin等^[68]^在利用IPRP-MS法分离肝素寡糖时，考察了离子对试剂丙胺（PPA）、三丙胺（TPA）、BTA、TBA和PTA在不同浓度下的离子对能力和分离性能等参数，其中PTA展现出出色的色谱性能，结合HFIP增强了质谱的响应。该工作利用IPRP-MS对亭扎肝素（一种酶解低分子量肝素）进行分析，结果如图6a所示^[68]^，较小聚合度寡糖有较好的色谱分辨率，随着寡糖聚合度增加，色谱分辨率显著下降。同时，较小聚合度但高电荷寡糖与相邻较大聚合度但低电荷寡糖有共洗脱现象，保留时间重叠，分离度较差。在MS数据处理时，除了大量多价态分子离子峰出现外，离子对试剂的加合物也大量出现，进一步增大了质谱解析的难度。整体数据呈现多价态、多加合的形式，因此在建立质谱数据库时应考虑加合物以及加合物存在下的多价态。

**图6:**
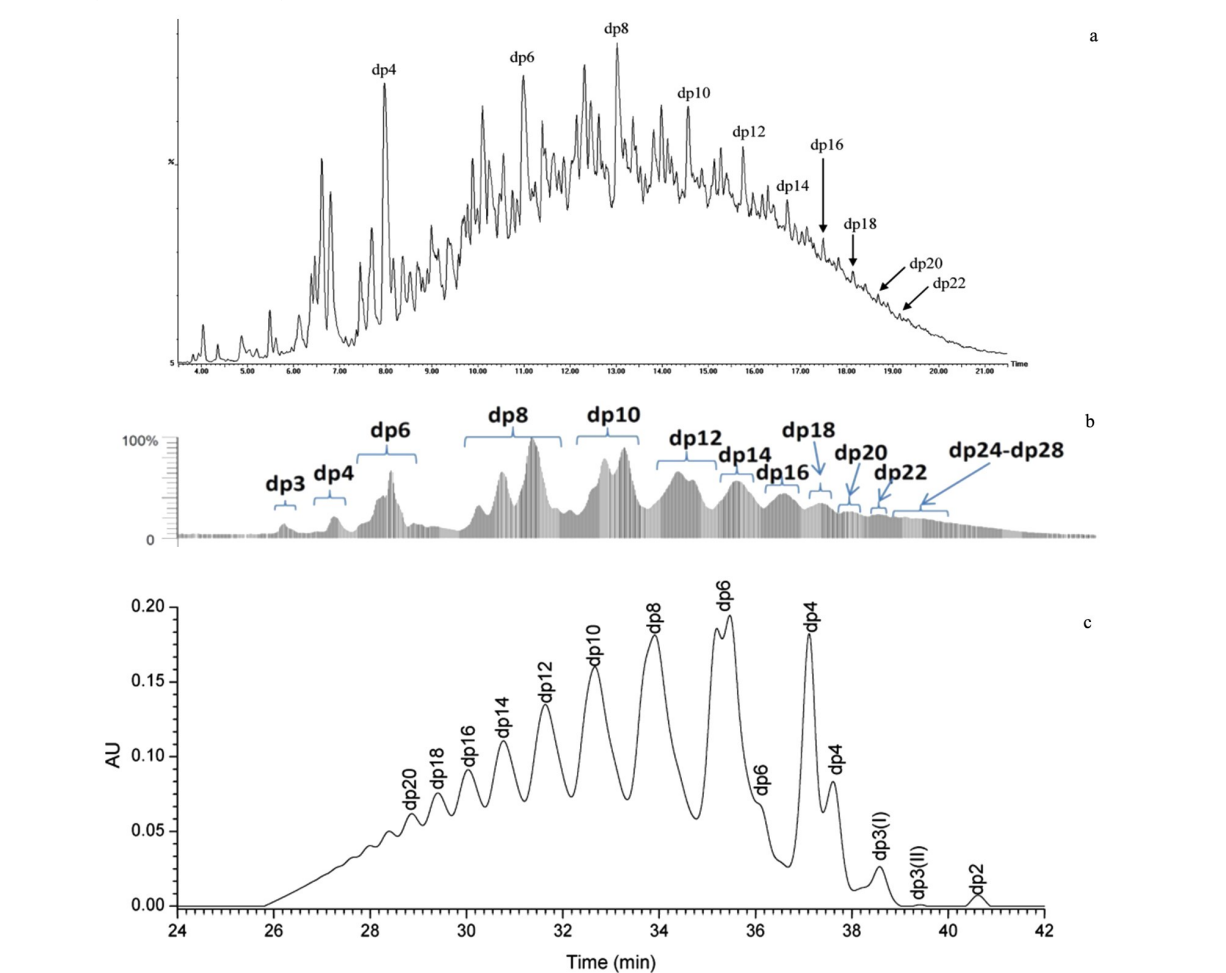
LMWHs的寡糖分析^[68,85,86]^

### 3.2 HILIC-MS

HILIC在分析肝素类药物寡糖的过程中由于流动相中盐和有机相梯度导致紫外检测有较高的背景，大多以质谱为检测手段。2012年Li等^[85]^采用HILIC-MS对依诺肝素钠进行了系统分析，结果如图6b所示。从结果可以看出，该方法相对于IPRP分辨率较低，稳定性较好，也没有离子对试剂残留的问题。该方法利用依诺肝素钠糖链组成的虚拟数据库，完成了依诺肝素钠糖链的质谱数据检索和归属，同时利用提取离子流对多个厂家的依诺肝素钠不同聚合度寡糖的组成进行了对比研究。HILIC-MS以较好的质谱兼容性将成为最有潜力的寡糖序列分析方法之一。

### 3.3 SEC-MS

相对分子质量和相对分子质量分布是肝素类药物重要的质量指标，SEC法作为测定肝素类药物相对分子质量和相对分子质量分布的法定方法收录在各国药典中^[54,62]^。这些方法往往结合示差检测或者多角度激光散射检测，前者需要一系列相对分子质量已知的单分散性聚合物作为标准品，并根据保留时间准确推断测试样品的相对分子质量；后者则根据不同角度激光散射的强度直接计算相对分子质量^[89]^。2017年Ouyang等^[90]^在此基础上，进一步探索了肝素类药物在SEC中的色谱行为，特别是研究者将SEC与电感耦合等离子体质谱（ICP-MS）在线连接，确定了肝素类药物中的阳离子在SEC色谱中被流动相中的阳离子替换，原有阳离子，如钠或者钙以盐的形式在接近总保留体积（*V*_t_）处流出。因此，以SEC方法分析肝素类药物的相对分子质量和含量时，阳离子的因素需要酌情考虑。

SEC有着极高的稳定性，同时按相对分子质量大小或者聚合度分离，符合肝素类寡糖结构特点。近年来，随着色谱材料的不断发展，超高分辨SEC技术在肝素类寡糖分析中得到了很好的应用。2012年康经武教授课题组^[86]^发表了超高分辨SEC-MS分析依诺肝素钠工作，如图6c所示，该方法使用了较低浓度的挥发性无机盐，质谱兼容性较好，色谱结果直观地体现了该低分子量肝素寡糖分布和组成比例。2019年Ouyang等^[59]^利用该方法完成了依诺肝素钠大样本量分析，区分了不同工艺、厂家、生产时间、种属来源的40余个样品。随着超高分辨SEC对低分子量肝素寡糖色谱分辨率的巨大提升，在线质谱对这些寡糖定性分析也有了长足的进步，然而，流动相中挥发性铵盐的多种加合物以及电喷雾电离产生的多价态导致质谱数据自动解析出现大量假阳性结果，无法摆脱大量繁复的人工解析工作。2016年Zaia教授^[91]^在此基础上，引入了在线阳离子抑制器，去除了阳离子加合物对质谱数据的影响；同时建立了一个针对肝素类物质糖链同位素质谱数据库，并以此数据库为基础，改善了肝素类物质糖链质谱数据中多价态导致去卷积过程中同位素信号识别错误引起的假阳性结果，实现了肝素类物质糖链的自动化质谱解析和归属。2022年Yan等^[92]^从另一个角度开发了一款基于色谱拟合和质谱归属校正的新型糖谱分析软件Glycomapping，这款软件首先通过特有的色谱拟合方法极大提高了SEC分离肝素类物质糖链的分辨率，每个聚合度对应的色谱峰，包括肩峰、隐藏峰均拟合成独立色谱峰，确定了准确的起始和结束时间点。之后，每个聚合度对应的色谱峰的起始保留时间进一步规范了质谱的自动化归属，过程中对于质谱去卷积错误导致的自动化归属错误，通过色谱保留时间自行矫正，并重新尝试多种价态去卷积并自动化归属，直至质谱自动解析与拟合色谱保留时间相匹配。该软件消除了去卷积错误产生的假阳性，实现复杂硫酸化多糖液相色谱-质谱联用数据的准确、自动化分析。

### 3.4 MHC 2D-LC-MS

目前传统的一维液相色谱在分析肝素类寡糖时，依然无法实现所有寡糖的基线分离，无法更准确地回答肝素类药物中糖链的数量、组成和结构序列。2015年Ouyang等^[93]^首次将MHC 2D-LC-MS应用于LMWHs寡糖指纹图谱分析中。在该方法中，有着出色稳定性的SEC作为第一维色谱，实现不同聚合度寡糖的分离，有着出色分辨率的IPRP作为第二维色谱，实现相同聚合度下不同组成序列结构寡糖的分离。该方法是目前LMWHs寡糖分析方法中分辨率最高、组分结构解析最全面的方法，其中依诺肝素钠有超过120种寡糖被物理分离并根据相应MS信息进行了归属，那屈肝素钙有超过80种寡糖被物理分离并归属（见图7）。该方法首次将二维液相色谱技术应用于肝素寡糖的分析，得到二维液相色谱领域资深科学家Stoll的认可^[94]^。MHC 2D-LC-MS为LMWHs的结构组成研究、质量控制和肝素相关糖组学研究提供有力分析工具。

**图7:**
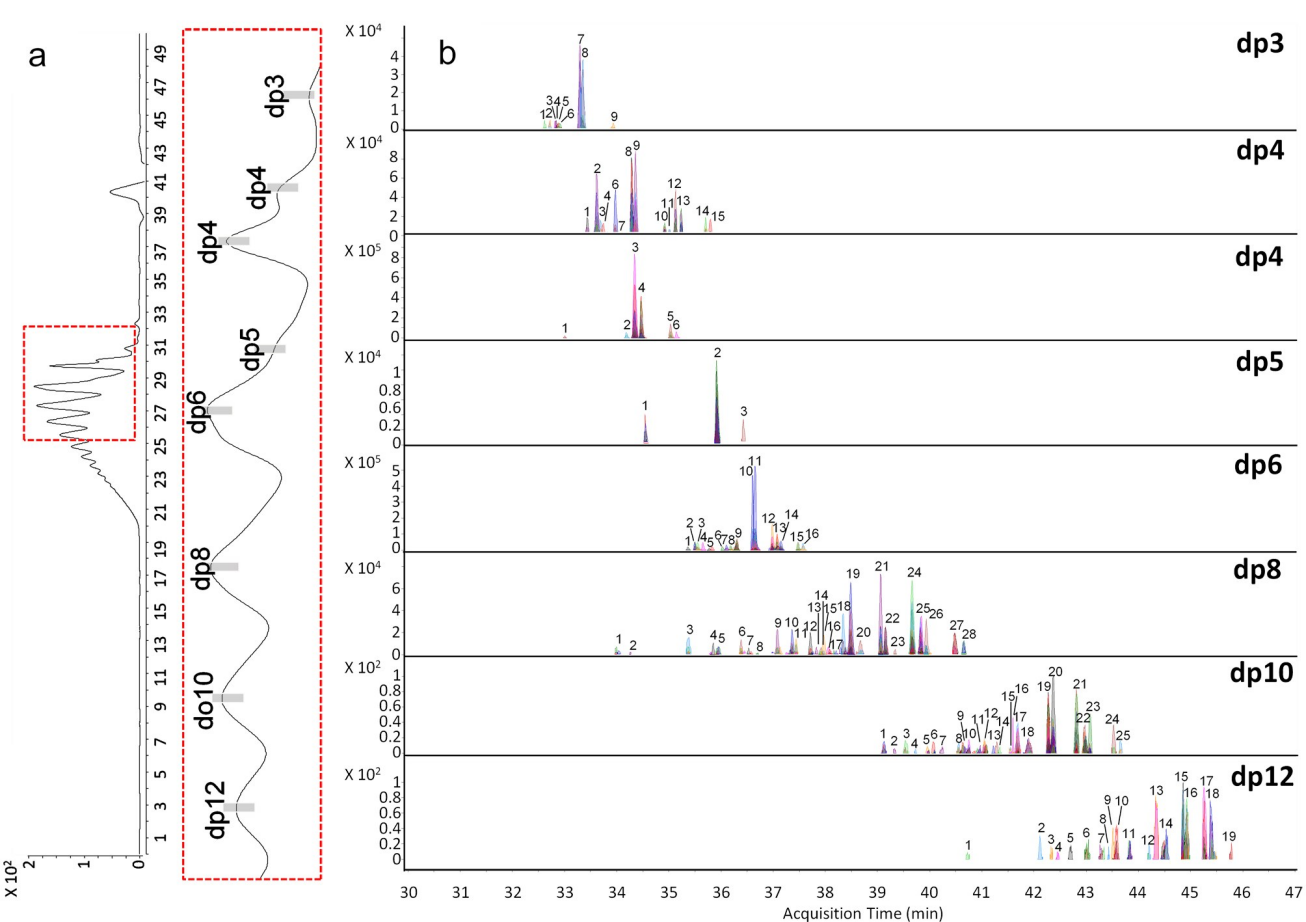
MHC 2D-LC-MS分析依诺肝素的寡糖图谱分析^[93]^

## 4 聚糖分析

肝素是相对分子质量较大的线性硫酸多糖，通过色谱方法直接对肝素聚糖进行分析，是一种更直观的分析策略。特别是2008年肝素污染事件之后，SAX作为法定方法对肝素中OSCS定性定量分析已被USP收录^[61]^。2009年Trehy等^[47]^通过建立的SAX-UV的方法将肝素、DS、OSCS基线分离并完成了定量分析，如图8所示，其中污染物OSCS的LOD和LOQ分别是0.03%和0.1%， DS的LOD和LOQ分别是0.1%和0.8%。

**图8:**
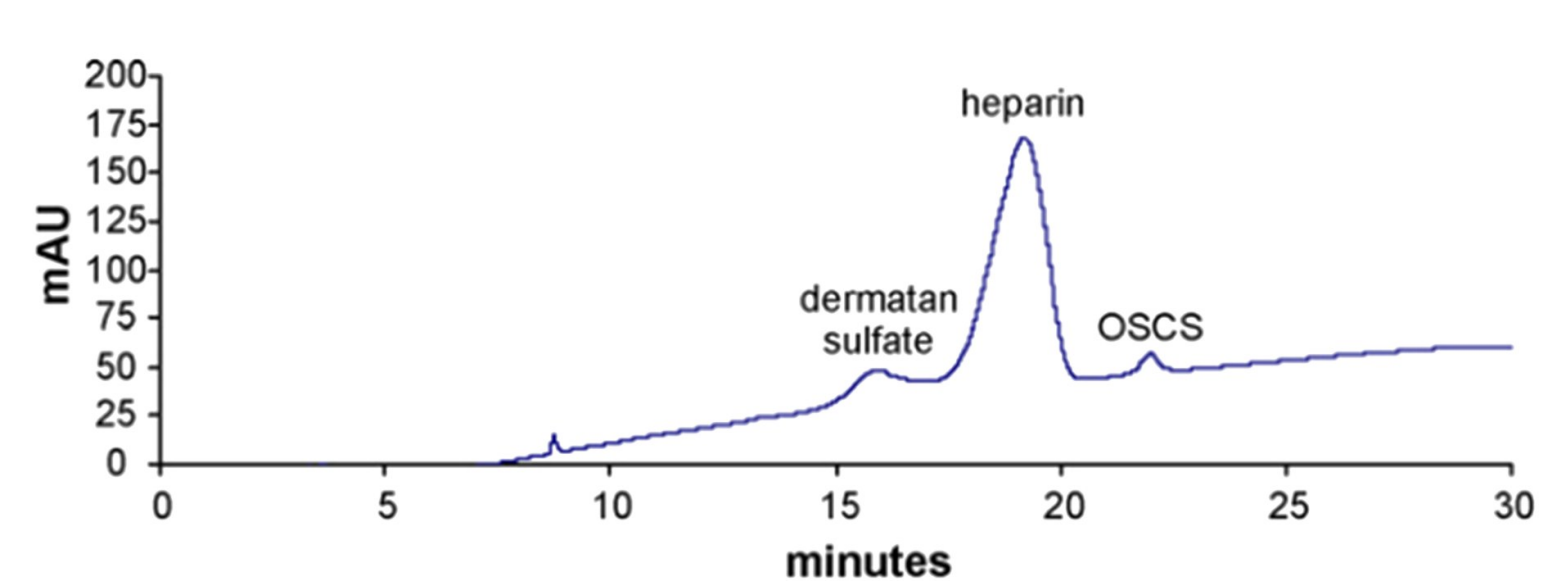
SAX-UV法测定肝素类似聚糖的色谱图（215 nm）^[47]^

## 5 结论

肝素类药物虽历经百年，但依然在临床上广泛应用，同时它又有多种潜在应用，在抗炎、抗肿瘤乃至抗病毒等领域都有着巨大的发展潜力。然而，肝素的结构异常复杂，结构解析、质量控制等相关的分析策略和方法一直是该领域的难点。多年来，肝素类药物结构序列分析主要集中在单糖组成分析、二糖组成分析、部分酶解寡糖产物序列结构分析、低分子量肝素寡糖组成序列结构分析以及聚糖分析等几个层次上。其中，单糖组成分析方法有一定的通用性，而其他方面的分析方法均相对独特，对仪器设备要求较高，技术难度相对较大。

SAX方法的开发历史较长，有着较好的稳定性，对UV检测适应性较好，有良好的拓展性，可以应用到二糖、寡糖和多糖的定性定量分析中。然而，流动相中高浓度的不挥发性盐使其无法与质谱联用，限制了其在发现新结构、定性分析低含量或特殊组分方面的应用。随着现代技术的发展，多维色谱技术使在线脱盐质谱联用得以实现，很大程度上推动了SAX在肝素分析应用上的发展。

IPRP方法最初用于核酸类物质的分析，在肝素类糖链分析中体现出了很好的色谱分辨率以及质谱兼容性。然而，IPRP试剂残留问题，以及UV检测的背景问题一定程度上限制了其应用范围，目前科研领域是其主要的应用场景。

HILIC方法色谱分辨率较好，围绕色谱填料的修饰和改造有较大的拓展空间。然而，该方法同样有着UV检测背景高的问题，目前主要是依赖质谱检测定性，应用场景主要集中在科研领域。

SEC方法有出色的色谱稳定性，更多的用在相对分子质量和相对分子质量分布测定上，但随着更加稳定的超高分辨色谱填料的出现，SEC方法在肝素类药物的寡糖分析方面展现出了极高的应用价值，具体体现在方法学稳定性好、色谱分辨率高、UV检测稳定性强、质谱兼容性强等方面。鉴于以上优点，很多肝素类寡糖自动化解析软件都是在SEC基础上开发应用的，在一定程度上也反映了该方法的优势和更广阔的应用前景。

CE法对分析较小聚合度寡糖有着高灵敏和高分辨率的特点。虽然其稳定性比色谱略差，但已经在科研和工业领域有着良好的应用。近些年来随着科技的发展，CE与质谱的兼容得以实现，这是CE在肝素类糖链分析应用中的巨大进步。然而，目前CE在与质谱兼容的过程中往往在一定程度上牺牲了分辨率，所以CE-MS更广泛的应用还有待技术的进一步发展。

以上不同的色谱分析方法都有各自的优势和弊端，二维液相色谱，可以兼具两种色谱的机制优势，实现肝素类药物结构序列更全面更细致的分析。基于二维液相色谱的肝素类糖链分析将是未来发展的一个重要方向。

总之，本文围绕肝素类药物组成和结构序列分析，从单糖、二糖、寡糖、聚糖4个维度介绍了不同色谱法的应用，并分别总结了它们的最新进展，希望对肝素类药物的质量控制以及结构序列分析有一定的参考价值。目前色谱技术正处于革新时代，相信随着色谱分离技术日新月异的发展，更多的方法和分析策略将应用于肝素类药物的组成和序列结构分析中，助力肝素类药物安全可控良性发展。
